# Polycomb Repressive Complex 2 (PRC2): A Context-Dependent Epigenetic Regulator of Brain Aging

**DOI:** 10.3390/biom16081175

**Published:** 2026-08-12

**Authors:** Maria A. Katsianou, Eleni-Kyriaki Vetsika, Mariam Markouli, Christina Piperi, Antonios N. Gargalionis

**Affiliations:** 1 Department of Biological Chemistry, Medical School, National and Kapodistrian University of Athens, 11527 Athens, Greece; makatsianou@med.uoa.gr (M.A.K.); cpiperi@med.uoa.gr (C.P.); 2 Department of Basic and Clinical Sciences, Medical School, University of Nicosia, 16777 Athens, Greece; vetsika.ek@unic.ac.cy; 3 Centre of New Biotechnologies and Precision Medicine (CNBPM), School of Medicine, National and Kapodistrian University of Athens, 11527 Athens, Greece; 4 Department of Medicine, Boston Medical Center, Boston University School of Medicine, Boston, MA 02118, USA; myriam.markouli@bmc.org

**Keywords:** Polycomb Repressive Complex 2, H3K27me3, brain aging, epigenetic modification, EZH2, PRC2-AgeIndex, neurodegeneration

## Abstract

Brain aging is characterized by extensive and dynamic epigenetic changes that reshape chromatin architecture and influence gene regulatory networks, leading to deregulation of transcriptional programs essential for neuronal survival, synaptic plasticity, and cognitive function. Emerging studies highlight the pivotal regulatory role of the Polycomb Repressive Complex 2 (PRC2) in brain aging, functioning mainly as an epigenetic silencer through the establishment of the H3K27me3 histone mark at gene promoters and enhancers, leading to transcriptional repression. Altered distribution and decreased activity of PRC2 during aging disrupts the balance between gene activation and repression, affecting pathways involved in neurogenesis, synaptic plasticity and stress response. Further interplay with other epigenetic regulators such as histone deacetylase 2 (HDAC2), DNA methyltransferases and non-coding RNAs forms an extensive network that contributes to cognitive decline, increasing vulnerability for neurodegeneration. In this review, we describe structural and functional aspects of PRC2, with emphasis on the potential of this epigenetic context-dependent regulator to integrate developmental, environmental and aging-related signals, to shape the transcriptional landscape of aging brain. We also discuss the newly developed PRC2-AgeIndex, which serves as a prognostic biomarker for age-related neurodegeneration and monitoring treatment response, as well as current PRC2-targeting options for healthy brain aging.

## 1. Introduction

Dysregulated brain function, also known as brain aging, contributes to an increased risk of developing neurodegenerative diseases by manifesting both cognitive impairment and behavioral changes [1]. It affects cellular and molecular pathways, being a complex process that affects the expression and activity of many genes [2]. The key hallmarks of aging involve transcriptional alterations such as the shortening of telomeres, genomic instability, cellular senescence, mitochondrial dysfunction, loss of proteostasis, and epigenetic alterations, as well as inflammation and oxidative stress [3].

Neuronal identity and its stable function depend on how well neural-specific genes respond to environmental signals, whose activity depends on tightly coordinated transcriptional and epigenetic mechanisms [2,4]. Proper brain development, including neuronal plasticity and cognitive function, is related to epigenetic mechanisms such as histone modifications, DNA methylation, and chromatin remodeling [4,5].

The Polycomb Repressive Complex 2 (PRC2) is a fundamental and evolutionarily conserved epigenetic regulator that establishes the trimethylation of histone H3 lysine 27 (H3K27me3), contributing to transcriptional repression across diverse biological contexts. Its functions range from regulation of embryonic development, lineage specification, stem cell maintenance, tissue homeostasis and cellular senescence to tumorigenesis [6]. PRC2-mediated chromatin regulation is particularly important in the nervous system for the control of developmental programs, maintaining neuronal identity, as well as regulating the cellular responses to environmental and stress signals. Aberrant PRC2 activity has been shown to affect neuronal homeostasis, neural differentiation, and brain function [7,8].

PRC2 activity changes during aging affecting the genomic distribution of H3K27me3 marks and altering the transcriptional programs involved in neuronal function and neurodegenerative disease [9,10,11,12]. Studies highlight that PRC2 interacts functionally with other epigenetic modifiers such as DNA methyltransferases (DNMTs) and histone deacetylase 2 (HDAC2). However, these interactions are highly dependent on the cellular context and have not been fully characterized in the aging brain [13,14,15]. PRC2 is also implicated in a wider network of epigenetic regulators, where modifications such as DNA methylation, histone deacetylation and chromatin accessibility impact transcription [16]. Elucidation of the mechanisms involved in PRC2-mediated epigenetic repression and the detection of potential alteration points during aging may contribute to developing novel strategies aimed at the preservation of brain function and delaying neurodegeneration. In this review, we focus on the emerging and context-dependent role of PRC2 in central nervous system (CNS) development and brain aging, while considering its functions within the broader biology of Polycomb-mediated epigenetic regulation. We further aim to connect PRC2 to neurodegenerative disease therapy, identifying potential molecular targets and therapeutic interventions.

## 2. Structural Aspects of the PRC2 Complex

The Polycomb-group (PcG) and Trithorax-group (TrxG) proteins were originally identified in *Drosophila melanogaster*, where during embryogenesis, they were shown to regulate the homeobox (*Hox*) genes [17]. These protein groups have antagonistic effects on PcG complexes, with PcG repressing transcription and TrxG complexes activating it [18,19,20].

The PcG family is composed of two major complexes, namely, PRC1 and PRC2, each composed of several core proteins [19]. PRC2 is a multi-subunit epigenetic regulator, highly conserved from *Drosophila* to mammals, and consists of four core proteins: the two lysine methyltransferase paralogs (EZH1 or EZH2), the suppressor of zeste 12 (SUZ12), embryonic ectoderm development (EED), and the histone binding proteins RB-Binding Protein 4 (RBBP4) or RB-Binding Protein 7 (RBBP7) [21,22,23]. EZH1 or EZH2 are the catalytic subunits which, by establishing H3K27me2/3 histone marks in neuronal and non-neuronal genes in developing neurons, contribute to transcriptional repression and cell fate regulation [24,25,26]. EED acts as an allosteric regulator by recognizing pre-existing H3K27me3 marks and enhances PRC2 activity. SUZ12 acts as a structural scaffold that stabilizes the complex by controlling the interactions between catalytic and regulatory regions. RBBP4/7 facilitates the engagement of nucleosomes by enabling histone and chromatin binding [27]. Structural cryo-electron microscopy studies have also shown that these subunits form a distinctive four-lobed structure in which EZH2 wraps around the WD40 β-propeller of EED. Moreover, SUZ12 and RBBP4/7 are involved in the organization of the whole architecture required for efficient chromatin binding and enzymatic activity [28,29].

Recruitment of PRC2 to chromatin is now understood to depend on its accessory assemblies. PRC2 assembles into two PRC2 homocomplexes, namely, PRC2.1 and PRC2.2, which are distinguished by specific accessory proteins [30]. PRC2.1 contains a single Polycomb-like protein (PCL), either PHD finger protein 1/19 (PHF1 or PHF19) or the metal response element binding transcription factor 2 (MTF2), together with elongin BC and Polycomb Repressive Complex 2-associated protein (EPOP) or one of the LCOR isoforms, i.e., PALI1 or PALI2. On the other hand, PRC2.2 contains the cofactors Jumonji and AT-Rich interaction domain 2 (JARID2) and AEBP2 [31]. These complexes function in concert to maintain and establish gene repression during neuronal progenitor differentiation [30] (Table 1). PCL proteins (PHF1, PHF19 and MTF2) have a central role in PRC2 chromatin recruitment and are vital subunits of the PRC2 complex. CpG-rich DNA areas are primarily recognized by MTF2, facilitating the recruitment of PRC2.1 as well as the respective sediments of H3K27me3, resulting in the inhibition of transcription. The contribution of each PCL is attributed to varied cellular context, with MTF2 acting as a primary CpG island sensor, whereas PHF1 and PHF19 modulate PRC2 catalytic activity and chromatin targeting [30].

**Figure 1:**
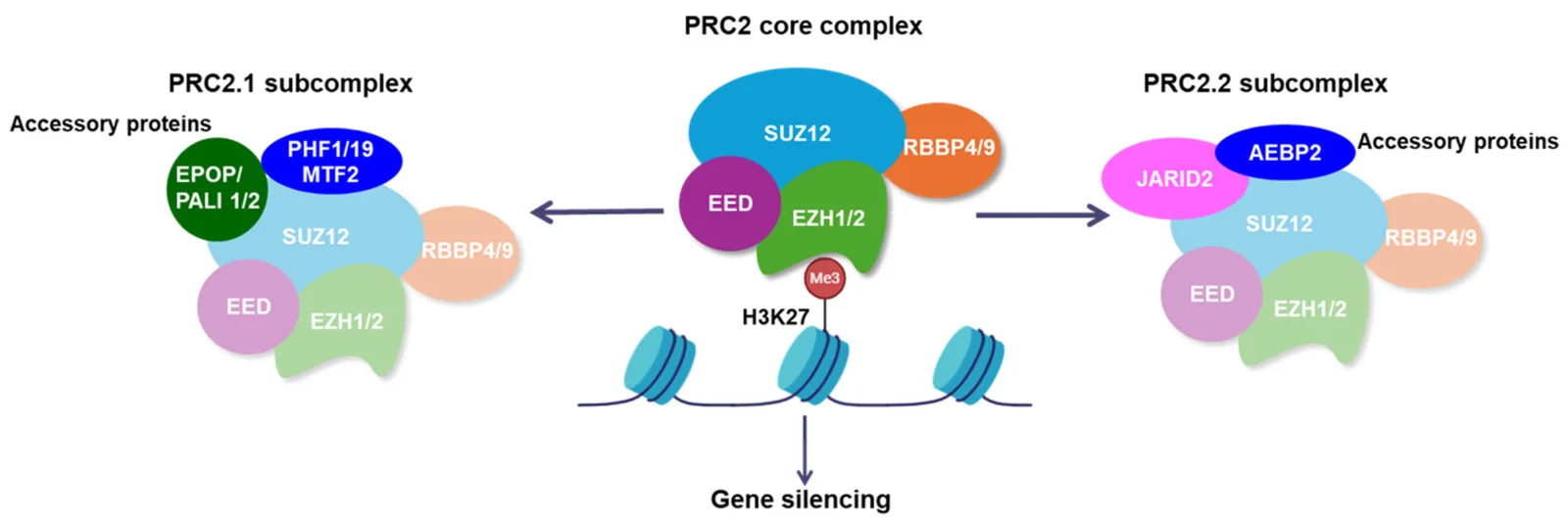
PRC2 core complex and subcomplexes. PRC2 is composed of the catalytic core unit, which includes EZH1/2, SUZ12, EED, and RBBP4 or RBBP7 proteins interacting with the accessory proteins PHF1/PHF19, MTF2, and EPOP or PALI1/2 to form the PRC2.1 subunit and JARID2 and AEBP2 to form the PRC2.2 subunit. PRC2 catalyzes the trimethylation of H3K27, resulting in gene silencing. EZH1/2, Enhancer of Zeste Homolog; SUZ12, Suppressor of Zeste 12 Homolog; EED, Embryonic Ectoderm Development; RBBP4/7, RB-Binding Protein 4/7; PHF, PHD Finger Protein; MTF2, Metal Response Element Binding Transcription Factor 2; EPOP, Elongin BC and Polycomb Repressive Complex 2-Associated Protein; PALI, PRC2-Associated LCOR Isoform; JARID2, Jumonji and AT-Rich Interaction Domain Containing 2; AEBP2, Adipocyte Enhancer Binding Protein 2.

Moreover, accessory proteins such as JARID2, the adipocyte enhancer-binding protein 2 (AEBP2), and PCLs associate with PRC2 and regulate its stability and enzymatic activity [25] (Figure 1). Among JmjC-domain proteins, JARID2 is primarily associated with PRC2 and functions as a core assembly factor of the PRC2.2 complex. In contrast, JMJD3 functions independently of PRC2 as an H3K27me3 demethylase and should not be considered a PRC2-associated protein. The zinc finger protein AEBP2, which commonly interacts directly with PRC2 [32,33], boosts its methyltransferase activity, promoting adipocyte migration and neural crest formation [34,35].

The activity of PRC2 can be regulated by chromatin features, the above-mentioned facultative subunits, and diverse post-translational modifications, including DNA methylation, histone modifications, and non-coding RNAs [11]. Several factors such as DNA methylation and histone modifications, especially the pre-existing activating histone marks H3K36me3 and H3K4me3, can modulate PRC2 binding. Additional post-translational modifications, including JARID2 methylation, phosphorylation, acetylation, ubiquitination, SUMOylation, and O-Glc-NAcylation, have been demonstrated to alter the assembly and stability of the complex, as well as its enzymatic activity, contributing to PRC2 dysregulation [19,36,37,38,39,40,41,42,43].

**Table 1:** The role of PRC2 core and accessory proteins in CNS development and brain aging.

	CNS Development	Aging Brain	References
**Core Subunit**			
**EZH1**	Catalyzing H3K27me3 Higher nucleosome/DNA binding activity relative to EZH2 Compact chromatin robustly	Maintains repressive chromatin in adult neurons	[24,44,45]
**EZH2**	Catalyzing H3K27me3 NPC proliferation, differentiation	PRC2-deficiency → neurodegeneration (e.g., HD) Impaired neural survival	[7,46,47,48,49]
**SUZ12**	Essential for PRC2 catalytic function Regulation of neurogenesis	Altered PRC2 activity	[46,50,51]
**EED**	Activation of PRC2 by H3K27me3 binding Regulates neurogenesis	Conditional PRC2-KO leads to neurodegeneration and premature neuron differentiation	[46,52,53,54]
**RBBP4/7**	Histone binding Not essential for catalysis but required for gene silencing	Participating in aging-associated epigenetic changes involving H3K27me3	[19,46]
**Accessory Proteins—PRC2.1**			
**PHF1/19** **MTF2**	Enhances PRC2.1 methyltransferase activity	Maintenance of neuronal identity	[46]
**AEBP2**	Enhances PRC2.2 methyltransferase activity	Changes might contribute to impaired neural crest migration and aging-related transcriptional dysregulation	[34,35]
**EPOP**	Accessory Subunit	Dysregulation may influence aging-related H3K27me3 changes	[46]
**PALI**	Enhances PRC2.1 methyltransferase activity	Affecting aging epigenome	[46]
**Accessory Proteins—PRC2.2**			
**JARID2**	DNA/chromatin Binding of PRC2.2	Loss impairs PRC2 targeting neural genes Affecting age-related epigenetic shifts	[16,30,55]
**AEBP2**	Enhances PRC2.2 methyltransferase activity	Changes might contribute to impaired neural crest migration and aging-related transcriptional dysregulation	[34,35]

## 3. PRC2 Complex Role in CNS Development

Studies have shown that PcG proteins can maintain the pluripotent state of embryonic stem cells (ESCs) by repressing transcription factors including *Fox* and *Sox* genes, as well as *Hox* genes, which control growth, thus preventing premature differentiation [56,57,58]. The silencing of *Hox* gene expression by PRC2 has been demonstrated to specifically promote the formation and expansion of anterior brain regions, while subsequent experiments with PRC2 deletion had no effect on posterior brain areas [59,60,61].

During neurogenesis, EZH2 present in proliferating cells, ESCs, and neural progenitor cells (NPCs) can regulate the timing and number of neurons produced, as well as the thickness of the cortical plate [7,49,62]. In mice, early *Ezh2* knockdown in the forebrain or midbrain enhances early differentiation of NPCs into neurons, impairing normal brain growth [7,49]. Additionally, *Ezh2* is critical for regional brain specification since its deletion represses the midbrain markers Pax3 and Pax7 while driving the ectopic expression of the forebrain-specific genes *Foxg1* and Pax6 [49]. *Ezh2*-null mice exhibit reduced H3K27me3 levels in both NPCs and differentiated neurons, accompanied by early activation of cortical neuron-specific genes like *Bcl11b*, *Myt1l*, *Mef2c*, and *Neurod6* [7]. Loss of H3K27me3 disrupts transcription factor networks in the ventricular zone [63]. In the developing cerebellum, conditional *Ezh2* deletion can upregulate GABAergic interneuron through *Pax2*, *Pax5*, and *Pax8* genes and downregulate the Purkinje cell markers (Rora, Olig1, Olig2), inducing cerebellar hypoplasia [63].

Within the subventricular zone, neurogenic astrocytes represent an astrocytic population with stem-like properties, which is implicated in the generation of neurons throughout adulthood [64,65]. The cells are distinguished from non-neurogenic astrocytes by high levels of EZH2 [64]. In neurogenic astrocytes, EZH2 suppresses the cell cycle inhibitors Cdkn2a or Ink4a/Arf to sustain astrocyte self-renewal, and can also repress the transcription factor Olig2, allowing for neuronal differentiation [64].

Developmental studies have shown that the PRC2 subunits, Eed and Suz12, are expressed in different brain regions during early embryonic development in both mice and humans [54]. Homozygous deletion of either *Suz12* or *Eed* leads to embryonic lethality at gastrulation [51,52]. However, heterozygous loss of *Suz12* allows for survival but leads to neural tube and brain malformations [50].

Among most PRC2’s major genomic targets are *Hox* homeotic genes, which regulate anterior–posterior brain patterning. Conditional deletion studies demonstrate that *Eed* is vital for both NSC proliferation and neurogenesis as its loss causes premature NSC differentiation while reducing neurogenesis. Notably, PcG repression prevents *Hox* homeotic gene activation in anterior and posterior body regions [66,67]. Respectively, mammals lacking the PRC2 protein Eed exhibit a loss of H3K27me3 marks in the embryonic CNS [61].

The core PRC2 protein, RBAP46/48, is a universal histone chaperone that contributes to chromosomal stability [68]. While RBAP46/48 is dispensable for PRC2’s enzymatic activity [69], its chromatin-remodeling capacity has been shown to facilitate the suppression of genes specific to NSCs by promoting chromatin compaction during differentiation [19] (Table 1).

## 4. Implication of PRC2 Activity in Brain Aging

Recent research studies have demonstrated the potential of PcG proteins in preventing neurodegeneration and brain aging by maintaining repressive chromatin states, silencing pro-apoptotic and senescence-associated genes, preserving neural stem cell pools, and supporting proper neuronal identity and function [19] (Table 1). PRC2, beyond its established function in development, plays an important regulatory role in the gene expression of neurons through the deposition of repressive histone marks H3K27me3 [70,71]. Altered PRC2 activity and H3K27me3 depositions have been reported in both physiological aging and neurodegenerative disease, suggesting that deregulated PRC2 activity may contribute to neurodegeneration [70,71]. Notably, recent studies indicate that PRC2 activity is not decreased per se, but is highly context-, cell-, tissue- and disease-dependent [16,72]. They highlight that PRC2 should not be considered simply either an upstream driver or a passive downstream consequence of brain aging; rather, the studies support a dynamic and bidirectional model, in which age-associated alterations in DNA methylation, chromatin accessibility, histone modifications, cellular stress, and other epigenetic regulators can modify PRC2 recruitment and activity, while subsequent PRC2 dysregulation can reinforce aging-associated transcriptional and functional changes [16,70,71,72]. Redistribution or loss of PRC2-mediated H3K27me3 repression may therefore promote the inappropriate activation of developmental, senescence-, apoptotic-, and stress-responsive genes, and increase neuronal vulnerability, which contributes to neurodegeneration.

### 4.1. PRC2 Recruitment and Chromatin Targeting During Brain Aging

An important unresolved question in brain aging is whether age-associated changes in PRC2 activity reflect altered abundance of the complex or changes in its chromatin recruitment. PRC2 targeting is determined not only by the core EZH1/2–SUZ12–EED complex but also by accessory factors that confer genomic specificity. As previously described, PCL proteins within PRC2.1, including MTF2, PHF1 and PHF19, can recognize CpG-rich regions and regulate both the PRC2 occupancy as well as its catalytic activity. Additionally, JARID2 and AEBP2 within PRC2.2 provide alternative mechanisms for chromatin association and enzymatic regulation [30,31]. These interactions provide a potential mechanistic link between age-associated epigenetic remodeling and changes in PRC2 function.

Studies have shown that PRC2 target regions are frequently enriched in CpG islands, which undergo substantial age-related changes in DNA methylation. Therefore, alterations in CpG methylation, chromatin accessibility, and pre-existing histone modifications, including H3K4me3 and H3K36me3, can influence PRC2 recruitment and the local deposition of H3K27me3 [30,31]. Importantly, current evidence does not support a uniform increase or decrease in PRC2 recruitment across the aging brain. It has been suggested that PRC2 occupancy is likely to be remodeled in a cell- and locus-dependent manner, potentially resulting in loss of repression of genes that require continued silencing or the inappropriate repression of genes involved in neuronal plasticity, stress responses, and inflammatory regulation.

The spatial restriction of PRC2 activity is particularly relevant to neuronal function. Integration of accessory-factor binding with local DNA methylation, chromatin accessibility, histone modifications, and transcriptional state is expected to constrain H3K27me3 deposition to defined chromatin domains while limiting inappropriate repression of neighboring genes that are required for cellular identity and survival. Whether aging disrupts these mechanisms and promotes the aberrant redistribution or spreading of PRC2/H3K27me3 still remains unknown. Cell type- and locus-resolved profiling of PRC2 occupancy and H3K27me3 in young and aged brains will therefore be essential for determining how PRC2 chromatin targeting changes during aging.

Of note, these potential age-related changes should not be interpreted as evidence that PRC2 is a brain-specific regulator. PRC2 performs conserved functions in development, differentiation, stem-cell maintenance, and tissue homeostasis across tissues. The distinctive consequences of its dysregulation in the brain may instead reflect the specialized biology of neural cells, particularly long-lived, predominantly post-mitotic neurons that must preserve stable cellular identity while maintaining the transcriptional plasticity required for synaptic function and adaptation. Therefore, altered PRC2 recruitment may contribute to neuronal dysfunction and neurodegenerative vulnerability while representing a tissue-specific manifestation of a broader epigenetic aging mechanism. Comparative, cell type-specific studies across tissues are further required to determine which aspects of PRC2 dysregulation are specific to the aging brain and which reflect conserved mechanisms of cellular aging.

### 4.2. Cell Type-Specific Functions of PRC2 During Brain Aging

The aging process affects the functionality of various types of neurons in several brain regions, notably in the hippocampus and the prefrontal cortex, which are crucial for memory storage and cognitive adaptability [73]. Neurons are known to be among the longest-living cell types in the body, primarily formed during embryonic and early postnatal stages. During aging, they accumulate epigenetic modifications such as the loss of heterochromatin, increased DNA methylation of synaptic genes, shifts in histone modifications, and changes in chromatin remodeling. These alterations can impair synaptic maintenance and neuronal flexibility, leading to dysfunctional neuronal networks [74].

Contrastingly, glial cells succumb to aging by exhibiting epigenetic changes that promote inflammation and reactive states [75]. In addition, astrocytes, due to stress-induced activation of cytokines, transform into reactive inflammatory phenotypes, leading to modified astrocyte functionality [76]. Similarly, microglia are epigenetically predisposed to exaggerated inflammatory responses, whereas oligodendrocytes accumulate histone modifications that impair their capacity to remyelinate [77,78]. Therefore, this epigenetic aging affects both neurons and glia cells, and along with protein aggregation, mitochondrial dysfunction, neuroinflammation, and cellular senescence promote neurodegenerative processes [1].

### 4.3. Chromatin Repression

Research data show that PRC2 can repress bivalent, cell cycle regulatory and apoptotic genes such as *Cdkn2a/b*, *Pmaip1* and *Igfbp3*. Moreover, several developmental transcriptional regulators such as E2F1, which promotes apoptosis upon activation, and ASCL1, a neuronal lineage regulator exhibiting a protective role against neurodegeneration, are regulated by PRC2 in mice [26]. Conditional PRC2 deficiency was shown to enable the ectopic de-repression of these genes, inducing neuronal vulnerability [26].

Furthermore, a decline in PRC2-mediated repression has been observed during aging that permits transcriptional access to previously silenced genes such as *Cdkn2a*, Cdkn2b, and other senescence- or apoptosis-associated genes [15]. Several studies suggest that decreased expression of the catalytic PRC2 subunit EZH2 results in the loss of complex stability, defective chromatin recruitment, derepression of PRC2 target genes, and decreased H3K27me3 [79,80]. This decline underlies the predominance of late-onset Alzheimer’s disease (LOAD), which accounts for 90–95% of cases. It remains unclear whether dysfunctional PRC2 primarily mediates neuronal aging or whether it is the main downstream consequence of other cellular changes. In this context, the risk of acquiring LOAD and developing neurodegenerative changes significantly rises when PRC2 function deteriorates with age and is unable to effectively control these genes [15,26]. This finding indicates that the PRC2 complex not only plays a crucial role in producing appropriate epigenetic changes in embryonic cells but also maintains healthy brain processes throughout an individual’s lifespan. In aging brains, a deviation of methylation levels in AD-related genes, *APP* and *Wt1*, was consistent with reduced PRC2 activity [15].

In addition, approximately 20–30% of the upregulated PRC2-target genes, such as *Twist1*, *Pou4f1/2*, *Nkx2-5*, *Hand2*, *Wt1*, *Tal1* and numerous *Hox* genes, as well as 50% of the downregulated PRC2-target genes in PRC2-deficient medium spiny neurons (MSNs), correspond to upregulated and downregulated genes in the brain of patients with Huntington’s disease (HD), respectively [26,81,82]. This overlap indicates that loss of PRC2 may be a shared mechanism among neurodegenerative diseases, rather than being a disease-specific phenomenon.

### 4.4. Regulation of Neural Stem Cells

Beyond its role in suppressing detrimental transcriptional processes in developing neurons, PRC2 also promotes the specification of adult neurons during differentiation by preventing neural cells from expressing genes of non-neural lineages [83]. Mechanistically, this is achieved through H3K27 methylation, which prevents transcription factors from binding to DNA at non-neural loci. *Ezh2* knockout in the neurons of adult mice induces morphological changes and cognitive impairments, suggesting that loss of PRC2 may impact cognition in long term [84]. Deletion of *Ezh2* in adult neural stem/progenitor cells leads to reduced progenitor proliferation and deficits in memory, spatial learning, pattern separation, and fear conditioning [85]. Moreover, in cortical progenitor cells, the loss of *EZH2* induced a shift from self-renewal towards differentiation and decreased the neuronal output, changing the timing of neurogenesis [7]. Furthermore, *Eed* loss has been shown to induce defects in the proliferation of neural stem cells and reduce the formation of neurons, leading to impaired neurogenesis [86].

### 4.5. Interaction with Non-Coding RNAs (ncRNAs)

Non-coding RNAs (ncRNAs), including microRNAs (miRNAs) and long non-coding RNAs (lncRNAs), represent additional regulatory factors of PRC2 function. They can modulate the expression of PRC2 core or accessory components, interact with PRC2 proteins, and in some contexts, influence its association with chromatin [72]. However, the extent to which RNA directly determines PRC2 recruitment or stability in vivo remains unclear. RNA–PRC2 interactions appear to be highly context-dependent, and RNA binding alone is not sufficient to establish PRC2 recruitment to chromatin [72]. Therefore, ncRNAs can be considered as potential modulators of PRC2 abundance, activity and genomic distribution rather than universal determinants of PRC2 targeting.

Evidence from neuronal and neurodegenerative models supports a functional relationship between ncRNAs and PRC2. In *Drosophila*, miR-34 regulates PRC2-associated components, including SUZ12 and PCL proteins, leading to reduced H3K27me3 deposition and derepression of neuroprotective genes [13]. In Alzheimer’s disease (AD), miR-138 is upregulated and targets EZH2, while experimental studies have linked miR-138-mediated EZH2 suppression to reduced H3K27 methylation and altered tau/Aβ-related pathways [87,88]. LncRNAs may similarly interact with PRC2 to regulate specific gene loci. For example, BDNF-AS and ANRIL have been associated with PRC2-dependent repression of BDNF and the CDKN2A/CDKN2B locus, respectively, and their expression is altered in aging and AD models [7,89]. These findings suggest that ncRNAs may provide an interface between age-associated changes in RNA expression and PRC2-dependent chromatin regulation.

Nevertheless, direct evidence for ncRNA-mediated, locus-specific PRC2 recruitment during physiological human brain aging remains limited. It is still unclear whether age-associated changes in individual ncRNAs primarily alter PRC2 component abundance, modulate the activity of specific PRC2 assemblies, or influence PRC2 occupancy at defined genomic loci. Integrating ncRNA profiling with cell type-specific PRC2 occupancy and H3K27me3 mapping in young and aged brains will be important for establishing how ncRNA–PRC2 interactions contribute to epigenetic remodeling and neurodegenerative vulnerability.

### 4.6. Enhancement of Epigenetic Aging

**Figure 2:**
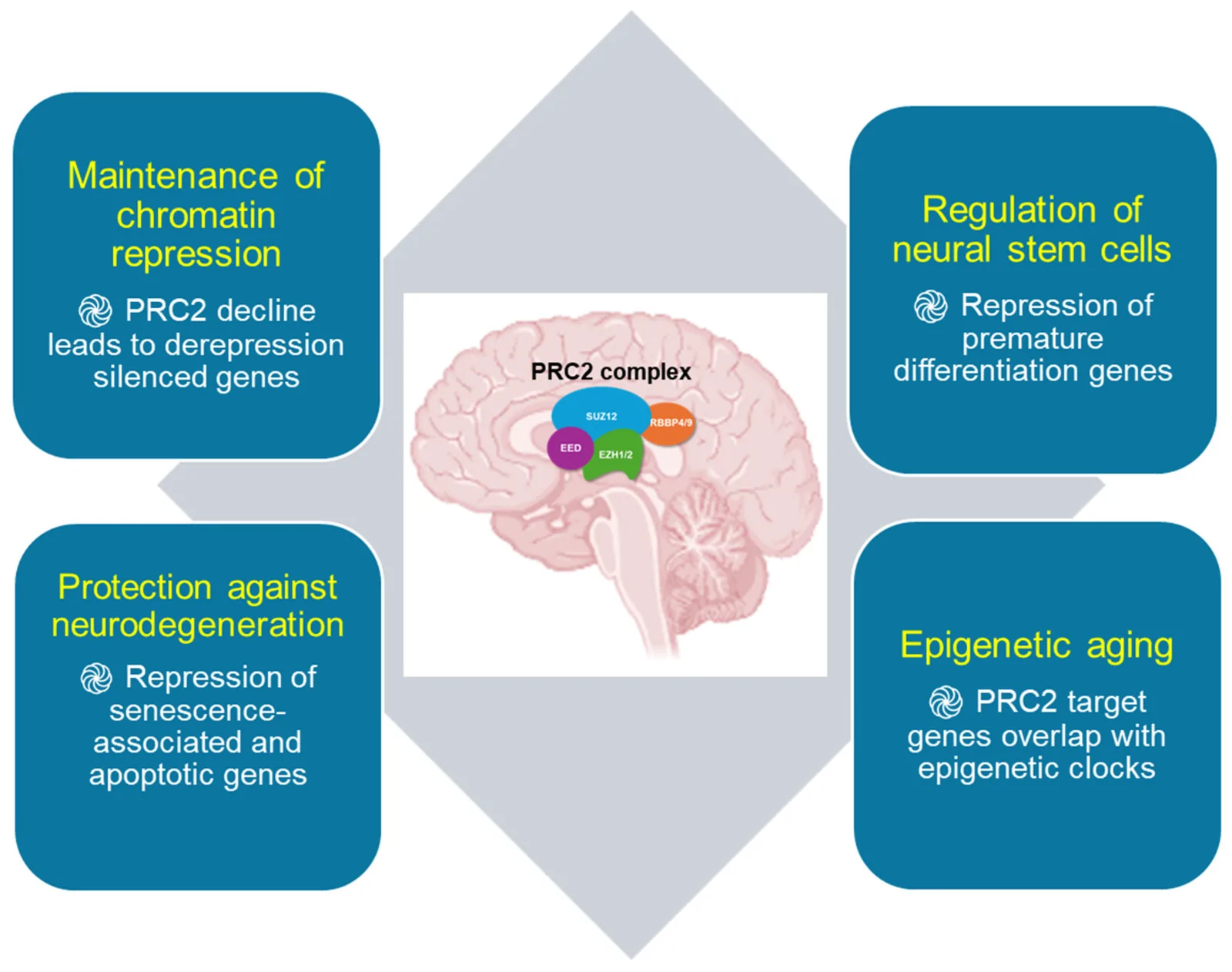
PRC2 activity in brain aging. PRC2 is involved in brain aging by maintaining repressive chromatin states, silencing pro-apoptotic and senescence-associated genes, preserving neural stem cell pools, and supporting proper neuronal identity and function.

Studies have shown that PRC2 target genes frequently overlap with CpG sites incorporated in age-associated epigenetic clocks, including Horvath’s clock and GrimAge, indicating that repression of these sites by PRC2 can maintain the youthful epigenetic patterns [90,91]. PRC2 loss of function of its target genes or their de-repression during aging may accelerate the onset of aging phenotypes. There is evidence that conserved CpG sequences, which consistently gain more methylation with age, including those near LHFPL3/4, appear to be located in PRC2-binding sites [9,92]. This further suggests that PRC2-mediated suppression can significantly influence the epigenetic regulation of aging and accelerate age-related neurodegenerative risk (Figure 2).

### 4.7. Translational Limitations of Current Evidence

Despite increasing evidence linking PRC2 to neuronal homeostasis and aging-associated phenotypes, the translational relevance of these findings to human brain aging are still under investigation. An important proportion of the mechanistic evidence supporting the beneficial effects of PRC2 modulation has been generated in lower organisms, particularly *Drosophila*, where manipulation of PRC2-associated pathways has been associated with enhanced neuronal resilience and protection from age-related degeneration. These studies provide valuable mechanistic insights but should not be directly extrapolated to human aging because of substantial interspecies differences in brain organization, cellular composition, lifespan, and epigenetic regulation. Mammalian models and studies of neurodegenerative disease provide complementary evidence that altered PRC2 activity and H3K27me3 deposition can influence neuronal survival, neurogenesis, inflammation, and disease progression. However, direct evidence that modulation of PRC2 promotes healthy brain aging in humans remains limited. Longitudinal, cell type-resolved studies in mammalian models and human brain tissue will be essential to establish whether age-associated changes in PRC2 activity are causally related to aging phenotypes and whether they can be safely manipulated therapeutically.

## 5. PRC2 as a Therapeutic Target

The therapeutic targeting of PRC2 in brain aging is promising and under extensive investigation since its effects are strongly dependent on cellular and disease context. Although excessive PRC2 activity and increased H3K27me3 have been associated with neuronal dysfunction and neuroinflammation in some disease models, PRC2 also maintains cellular identity and genome stability in multiple tissues, and loss of PRC2 function can promote oncogenic transformation in specific cellular contexts [93,94]. Thus, systemic or prolonged inhibition of PRC2 may have undesirable consequences, particularly in older individuals who already have increased susceptibility to cancer. Moreover, indiscriminate enhancement of PRC2 activity may also be detrimental if excessive H3K27me3-mediated repression suppresses genes required for neuronal survival or adaptive responses. The therapeutic goal should therefore be the restoration of an appropriate, context-dependent level of PRC2 activity rather than generalized inhibition or activation.

Evidence from neurodegenerative models provides a rationale for exploring this possibility. In Huntington’s disease (HD) mouse striatal neurons, reduced H3K27me3 and altered PRC1/PRC2 composition are accompanied by reactivation of developmental genes, including *Cbx4*, *Cbx8*, *Pax6*, *Onecut1* and *Runx2*, suggesting that loss of Polycomb-mediated repression contributes to disease-associated transcriptional instability [95]. Experimental studies indicate that modulation or stabilization of PRC2 activity can influence neuronal survival and inflammatory responses [96,97]. These observations indicate that restoring appropriate PRC2-dependent repression may be beneficial in selected neurodegenerative contexts, although whether such effects can be translated into safe therapeutic interventions remains unknown [98,99,100,101,102].

**Figure 3:**
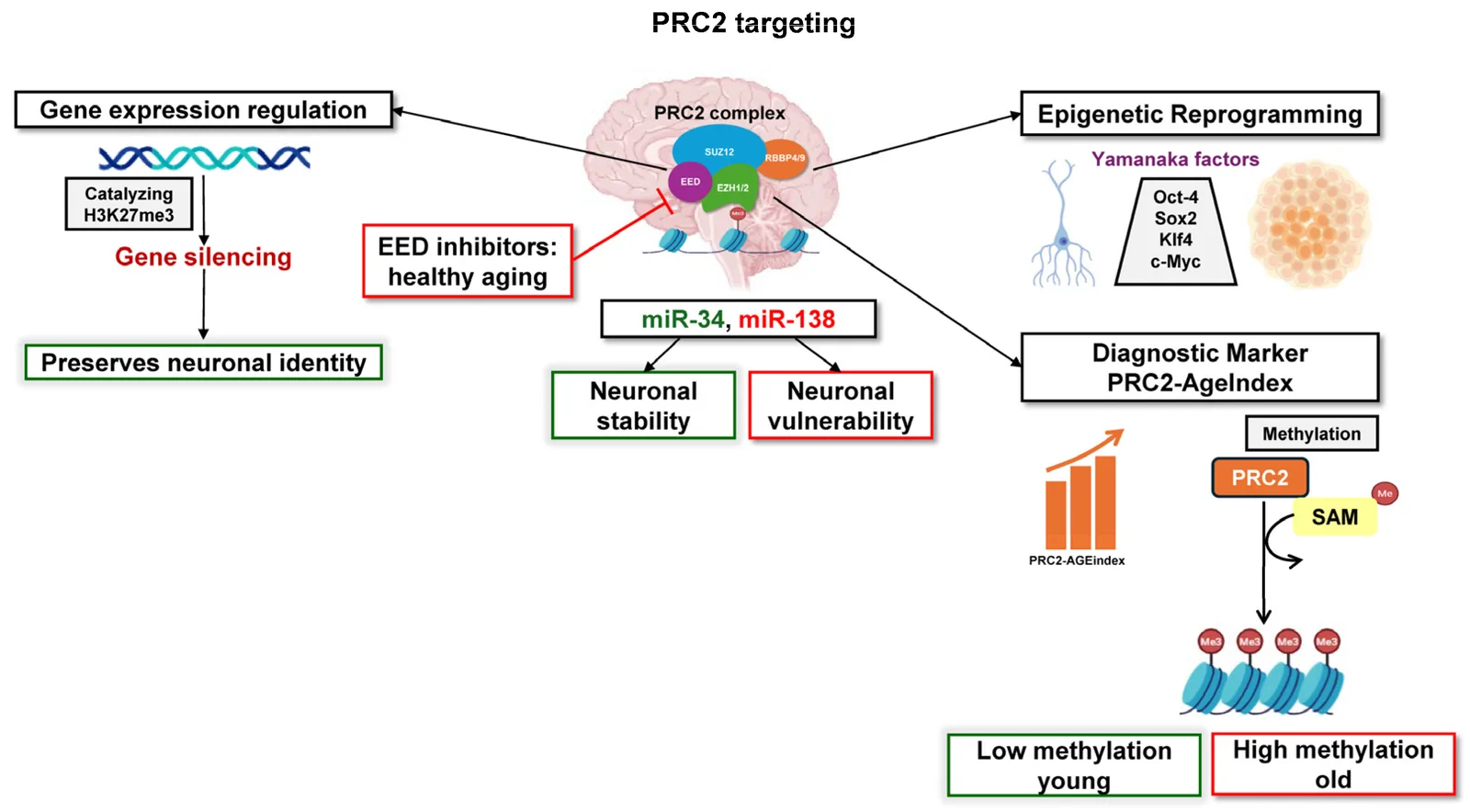
PRC2 can be targeted through small molecule inhibitors (EED, EZH2 inhibitors), epigenetic reprogramming, ncRNA modulation, and stem cell-focused interventions.

Pharmacological studies have primarily focused on EZH2 and EED because of their established roles in cancer. EED inhibitors, such as EED226 and MAK683, disrupt the allosteric activation and propagation of EZH2-mediated H3K27me3, whereas EZH2 inhibitors, including DZNep, GSK126, and tazemetostat, reduce PRC2 catalytic activity [103,104,105,106]. These compounds have provided important mechanistic insights, and in several experimental models of neuroinflammation, EZH2 inhibition has produced neuroprotective effects that depend on disease stage and cellular context [13,107,108,109]. However, most pharmacological evidence is still derived from oncology, and direct evidence supporting EED or EZH2 inhibitors as interventions for physiological brain aging is currently lacking. Their potential benefits therefore need to be weighed against the possibility that excessive PRC2 inhibition could compromise protective epigenetic programs in non-neural tissues (Figure 3).

Beyond direct pharmacological inhibition, alternative strategies may allow for more selective modulation of PRC2-associated pathways (Table 2). Epigenetic reprogramming using Yamanaka factors has shown rejuvenating effects in aged neural tissue and improved pathological and cognitive phenotypes in experimental Alzheimer’s disease models [110,111]. However, approaches involving pluripotency factors carry substantial safety concerns, including tumor formation associated with c-Myc, highlighting the challenges of translating broad epigenetic reprogramming into clinical applications [112]. Similarly, ncRNAs may provide an additional means of modulating PRC2 components. For example, miR-34 regulates SUZ12 and PCL proteins in *Drosophila*, whereas miR-138 targets EZH2 and has been implicated in AD-related chromatin dysregulation [13,87]. Although these findings are mechanistically informative, their relevance to human brain aging remains to be established.

An important priority for future therapeutic development is therefore the achievement of spatial and temporal specificity. Brain-selective delivery, cell type-specific modulation, and locus-specific epigenetic approaches could potentially limit systemic effects while preserving essential PRC2 functions in peripheral tissues. Such strategies may be complemented by biomarkers that identify abnormal PRC2 activity and monitor treatment response [113]. The recently developed PRC2-AgeIndex, based on age-associated DNA methylation changes at PRC2 low-methylated regions (LMRs), represents a potential biomarker of PRC2-associated epigenetic aging and has shown responsiveness to epigenetic interventions [9,114]. However, whether the PRC2-AgeIndex can reliably monitor therapeutic reversal of brain aging remains to be determined.

Overall, PRC2 should currently be considered as a potentially actionable but highly context-dependent regulator rather than an established therapeutic target for brain aging. Future studies should determine whether specific PRC2 alterations are causal in neuronal aging, identify the cell types and genomic loci in which modulation is beneficial, and establish whether brain-restricted interventions can achieve neuroprotection without compromising PRC2-dependent functions elsewhere or increasing oncogenic risk. Long-term safety studies in mammalian models and validation in human tissues will be essential before PRC2-directed interventions can be considered for clinical translation.

## 6. Future Perspectives

The PRC2 complex undoubtedly plays a vital role in neuronal regulation processes, and its dysregulation can be associated with brain aging. It has been demonstrated that PRC2 is recruited to target genes by the coordinated activity of its accessory components [115]. However, a detailed analysis of PRC2 recruitment to particular gene loci during aging and disease progression could elucidate its activity as well as any additional target genes. Since PRC2 is involved in both developmental processes and tumor suppression, understanding how it is recruited to specific genes may explain the reason its levels are dysregulated in malignancies and neurodegenerative diseases.

At the same time, the risks of tumorigenesis and effects of prolonged PRC2-therapies must be assessed and mitigated [116]. Thus, a thorough, comprehensive study of the PRC2 complex, including genomic recruitment predictability analysis, key factors influencing PRC2 upregulation and downregulation, and additional understanding of the distinct roles of PRC2 accessory proteins, will be extremely beneficial in developing novel treatment strategies against various age-related diseases.

Future therapeutic strategies targeting PRC2 in brain aging should prioritize brain- or cell type-selective modulation, rather than systemic manipulation, with the goal of preserving essential PRC2 functions in peripheral tissues and minimizing potential oncogenic risk [47,117]. Further to PRC2-AGEindex, more reliable biomarkers and indexes reflecting the PRC2 activity or epigenetic changes in peripheral blood should be produced so as to individualize treatment and enhance therapeutic efficacy [9,104]. Epigenetic tools such as CRISPR/dCas9-based tools may also provide opportunities for regulating epigenetic homeostasis and preserving chromatin structure [118]. Finally, combined therapies with anti-inflammatory agents and PRC2 inhibitors may simultaneously target brain aging and reduce neuroinflammation [119].

## 7. Conclusions

PRC2 has emerged as a central context-dependent epigenetic regulator in the brain whose activity is shaped by cell type, chromatin landscape, and interactions with accessory proteins, non-coding RNAs, and other epigenetic mechanisms. Modulation of PRC2 represents a promising therapeutic avenue, and successful clinical translation will require precise brain- and cell type-specific strategies that preserve the essential physiological functions of PRC2 and minimize systemic adverse effects, including the potential risk of tumorigenesis. A deeper understanding of the dynamic regulation of PRC2 in the aging brain will be essential for developing safe and effective epigenetic interventions against neurodegenerative disease.

## Figures and Tables

**Table 2:** PRC2 therapy targets in the aging brain.

Mechanism/Target	Mechanism of Action	Observed Effect	Model/Context	Therapeutic Implication	References
**PRC2-mediated H3K27me3**	Catalyzes H3K27 trimethylation→ repression of apoptotic genes	Neuronal gene expression: preserved	HD mouse striatal neurons	Stabilize neurons, limit neurodegeneration	[70,97]
**PRC1/PRC2 complex composition**	chromatin repressed by alterations in subunit ratios	Lower H3K27me3, reactivation of developmental genes	Huntington’s disease	PRC2 stabilization: maintain neuronal homeostasis	[70]
**miR-34**	Downregulates SUZ12 and PCL → reduces H3K27me3 → derepresses neuroprotective genes	Enhanced expression of neuroprotective chaperones; neuronal stability	*Drosophila* neurons	Upregulation reduces neurodegeneration risk	[113]
**miR-138**	Targets EZH2 → impaired H3K27me3 deposition → altered chromatin remodeling	Neuronal vulnerability, impaired gene silencing	Alzheimer’s disease models, tau phosphorylation	Targeting miR-138 may restore chromatin function	[87]
**EED inhibitors/PRC2 subunit stabilization**	Enhance PRC2 activity or stabilize chromatin binding	Promotes neuronal homeostasis and healthy aging	Neural tissue aging models	Pharmacological enhancement could delay neurodegeneration	[96]
**Epigenetic reprogramming (Yamanaka factors)**	Reactivates pluripotency genes → restores youthful epigenetic state	Rejuvenates aged neural tissue	Aged neural tissue	Potential rejuvenation therapy; c-Myc teratoma risk must be managed	[112]
**PRC2-AgeIndex**	DNA methylation changes at PRC2 LMRs accumulate with age	Universal marker of tissue aging	Multiple tissue types	Quantitative measure of therapy effectiveness; responsive to interventions	[9]
**EZH2 inhibitors**	Block EZH2 catalytic activity → reduce H3K27me3 deposition	Reduces brain inflammation; modulates gene repression	Inflammation models	Control neuroinflammation while preserving neuronal function	[13,107]
**PRC2 catalytic activity/subunit interdependence**	Regulates histone methylation and transcriptional silencing; impacts ECM, stress response	Alters cell survival, stress resistance	Cancer and aging brain models	Therapeutic targeting of EZH2, EED, or PROTAC-mediated degradation; overcome resistance; modulate tumor microenvironment	[99,102,103]
**Tazemetostat (EZH2 inhibitor)**	Selectively inhibits EZH2 → reduces H3K27me3	Modulates PRC2-dependent gene silencing	Cancer models	Demonstrates potential and challenges of PRC2 inhibition; resistance may develop	[98]

PRC2, Polycomb Repressive Complex 2; EZH1/2, Enhancer of Zeste Homolog; HD, Huntington’s Disease; PCL, Polycomb-Like Proteins; LMR, Low-Methylated Regions; PROTAC, Proteolysis Targeting Chimera.

## Data Availability

No new data were created or analyzed in this study. Data sharing is not applicable to this article.

## Abbreviations

The following abbreviations are used in this manuscript:

AD	Alzheimer’s disease
AEBP2	Adipocyte enhancer-binding protein 2
CNS	Central nervous system
EED	Embryonic ectoderm development
EPOP	Polycomb repressive complex 2-associated protein
ESC	Embryonic stem cell
EZ	Enhancer of zeste
HD	Huntington’s disease
HDAC2	Histone deacetylase 2
Hox	Homeobox
JARID2	Jumonji and AT-rich interaction domain 2
LMRs	Low-methylated regions
LOAD	Late-onset AD
miR-	microRNA-
MSNs	Medium spiny neurons
MTF2	Metal response element binding transcription factor 2
NPC	Neural progenitor cell
PALI1/2	PRC2-associated LCOR isoform 1 or 2
PcG	Polycomb-group
PHF	PHD finger protein
PRC	Polycomb repressive complex
PROTAC	Proteolysis targeting chimera
RBBP	RB-Binding Protein
Suz12	Suppressor of zeste 12
TrxG	Trithorax-group
Wt1	Wilms tumor 1
